# Increase lipid tear thickness after botulinum neurotoxin A injection in patients with blepharospasm and hemifacial spasm

**DOI:** 10.1038/s41598-018-26750-5

**Published:** 2018-05-30

**Authors:** Ren-Wen Ho, Po-Chiung Fang, Tsai-Ling Chao, Chun-Chih Chien, Ming-Tse Kuo

**Affiliations:** 1 Department of Ophthalmology, Kaohsiung Chang Gung Memorial Hospital and Chang Gung University College of Medicine, Kaohsiung, Taiwan; 2 Graduate Institute of Clinical Medicine, College of Medicine, Kaohsiung Medical University, Kaohsiung, Taiwan; 3 Department of Laboratory Medicine, Kaohsiung Chang Gung Memorial Hospital and Chang Gung University College of Medicine, Kaohsiung, Taiwan

## Abstract

The aim of this study was to investigate changes in the tear film lipid layer thickness (LLT) and aqueous tear production after botulinum neurotoxin A (BoNT) injection in patients with benign essential blepharospasm (BEB) and hemifacial spasm (HFS). Eleven and six patients with BEB and HFS, respectively, who received BoNT injection were consecutively enrolled in this prospective study. The blepharospasm disability index (BSDI), blink pattern, dry eye symptoms, Schirmer test 1 findings, LLT, eyelid performance, and corneal integrity were evaluated before and after treatment. Both BEB and HSF patients experienced remarkable relief from spasms and ocular discomfort after BoNT injection. LLT, the partial blink rate, the snap-back time, the lid distraction distance, and lateral canthal laxity were significantly increased at 1 month after treatment. There were no significant changes in Schirmer test 1 findings and meibomian gland dropout. Our findings suggest that LLT, a decisive factor for tear film stability, significantly increases at 1 month after BoNT injection for BEB and HFS. A decrease in BSDI and an increase in the snap-back time may contribute to the increase in LLT; this mechanism is probably responsible for the relief from dryness after BoNT injection in patients with facial movement disorders.

## Introduction

Benign essential blepharospasm (BEB) and hemifacial spasm (HFS) are associated with overactivity of the facial nerve and are the two most common facial movement disorders^1^. BEB is characterized by chronic, bilateral, constant, uncontrollable, and forcible contraction of the orbicularis oculi muscle, which results in functional blindness and affects the performance of daily activities by the patient^2^. HFS is characterized by unilateral, episodic, synchronized contractions of the facial musculature all the time, even during sleep, which is different from BEB. The prevalence rates for BEB and HFS are approximately 5 and 1 per 100,000 individuals, respectively^3–5^. HFS is primarily due to facial nerve compression by an aberrant or sagging arterial branch^2^, and approximately 1% cases are attributed to a tumour within the cerebellopontine angle or facial nerve trauma^6^. On the other hand, the exact cause of BEB remains unknown. In addition, patients with BEB may suffer from not only motor symptoms but also non-motor symptoms such as sensory problems, neuropsychiatric abnormalities, and even cognitive deficits, which can dramatically impair their quality of life^7,8^.

BoNT is an exotoxin produced by the bacterium *Clostridium botulinum* (an anaerobic, gram-positive bacillus)^9^. It blocks the release of acetylcholine at the neuromuscular junction of cholinergic nerves, causing temporary paralysis of the target muscles. Although several studies have demonstrated the long-term efficacy and safety of BoNT used for various ophthalmic disorders and cosmetic procedures^2,10–17^, unexpected chemodenervation of adjacent striated muscles has many potential complications, including ptosis, diplopia, lagophthalmos, eyelid malposition, and dry eye disease^2,18–24^.

Dry eye disease is the most common complication of BoNT injection for BEB and HFS, but the reported incidence greatly varies from 7.5% to 70%^19–22,24^. Besides, there are a debate for the effect of BoNT injection on dry eye disease; some researchers reported this treatment may worse the tear film stability but others claimed BoNT injection could be an alternative treatment for dry eye disease^25–27^. Meibomian gland dysfunction is the leading cause of dry eye disease; it causes change of lipid tears, makes tear film unstable, and contributes to rapid tear evaporation. However, most studies on post-BoNT dry eye disease are based on evaluations of aqueous tear production and tear drainage^16,28,29^. Functional changes in the meibomian glands and alterations in the tear film lipid layer thickness (LLT) after BoNT injection for BEB and HFS remain unclear. Therefore, the aims of this study were to assess the changes in the tear film LLT after BoNT injection and explore the association between LLT changes and characteristic BoNT sequelae in patients with BEB and HFS.

## Materials and Methods

### Subjects

This prospective case series, which was part of an investigation of ocular adnexal microorganisms, included patients with BEB or HFS who were treated with the commercialized BoNT, onabotulinum toxin A (Botox^®^, Allergan, Inc., Irvine, CA), at the oculoplastic department of Kaohsiung Chang Gung Memorial Hospital (CGMH) between 1 February 2016 and 30 June 2016. Informed consent was obtained from each subject, and all procedures adhered to the Declaration of Helsinki and the ARVO statement on human subjects. Institutional Review Board/Ethics Committee approval was obtained from the Committee of Medical Ethics and Human Experiments of CGMH, Taiwan.

Patients who underwent ocular or eyelid surgery within 1 year of the injection; those who received neuroleptic agents or drugs that interfere with neuromuscular transmission (aminoglycosides, calcium channel blockers, penicillamine), BoNT injection within the past 4 months, topical eye drops except for dry eye medications, artificial tears within 4 h or lubricating ointment within 12 h before ocular examination, and/or medications that could react with BoNT injection; those with allergy to any ingredients in Botox^®^; those with local infection at the injection site; pregnant patients; and those with diseases involving neuromuscular junctions were excluded. One eye of each BEB patient was randomly recruited using Bernoulli distribution. Only the diseased eye of each HFS patient was recruited.

### Treatment protocols and data collection

**Figure 1:**
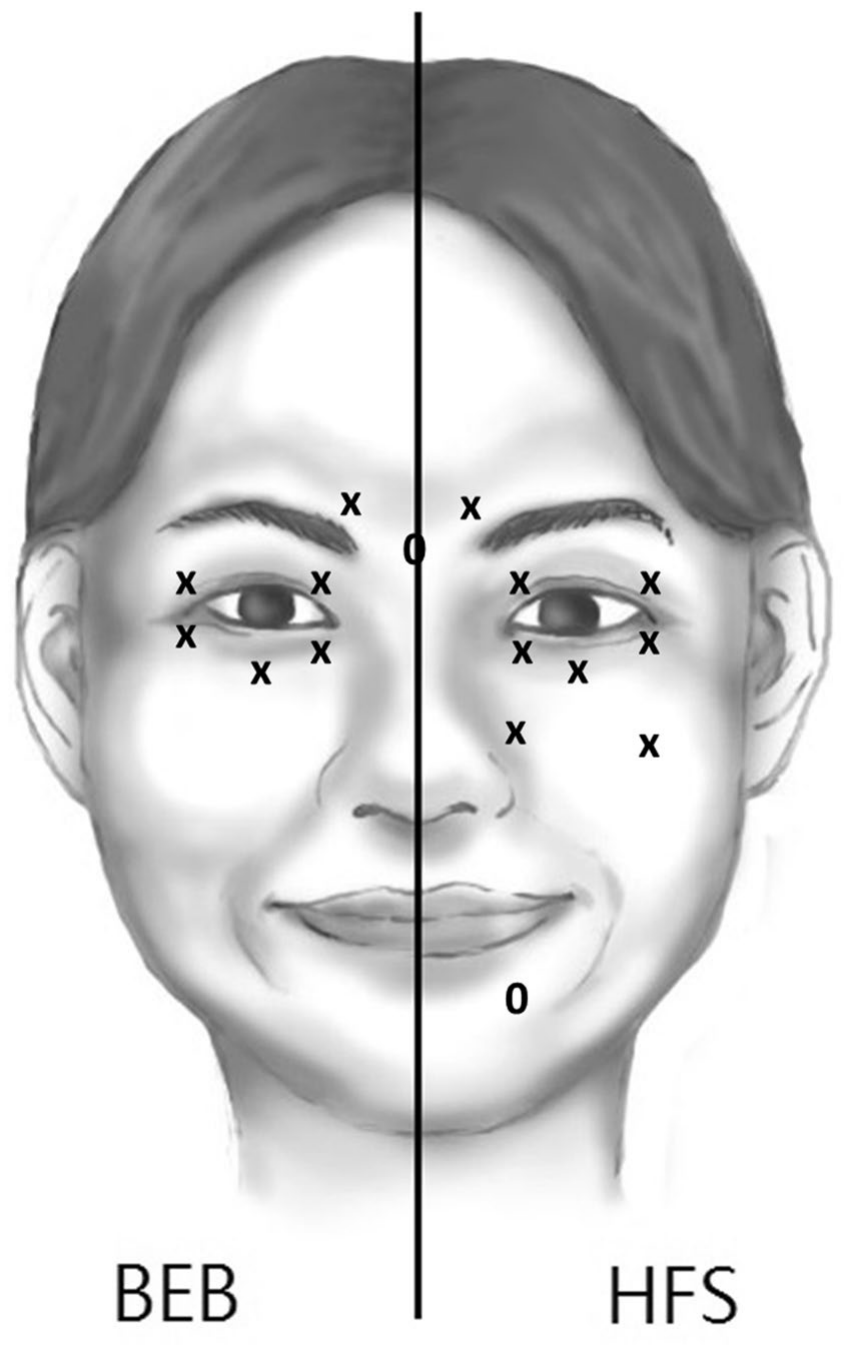
The injection sites scheme for the treatment of benign essential blepharospasm (BEB, left part) and hemifacial spasm (HFS, right part). The dose was 2.5 units per point. For BEB, 12-13 periocular points, 6 on each side with/without 1 procerus muscle, are injected. For HFS, 6-7 periocular points and 2-3 facial points on affected site are injected. ‘x’ means fixed injection point and ‘o’ means elective injection point.

Each vacuum-dried Botox^®^ was reconstituted with 2 mL of sterile, preservative-free normal saline to achieve a concentration of 5 units per 0.1 mL. All injections were administered by a single ophthalmologist (RWH). For patients with BEB, the doses of Botox^®^ were 15–17.5 units per eye, injected just beneath the skin. There were 6–7 injection sites, including the medial and lateral portions of the upper and lower pre-tarsal orbicularis oculi muscles, central portion of the lower pre-septal orbicularis oculi muscle, and corrugator muscle with or without the procerus muscle (Fig. 1, left). For patients with HFS, total 20–25 units of Botox® were administered, including 15–17.5 units to orbicularis oculi muscle and corrugator muscle and 5–7.5 units into the lower affected facial muscles (Fig. 1, right).

In additional to the clinical profiles of patients and the BoNT doses, the following parameters were evaluated before and after BoNT treatment: blepharospasm disability index (BSDI), representative ocular symptoms of dry eye disease (dryness, irritation, tearing), aqueous tear production, meibomian gland function (tear film LLT), meibomian gland morphology (dropout), blink rates and patterns, eyelid performance (eyelid laxity, lagophthalmos, scleral show, and ectropion), and corneal integrity (punctate erosion).

### Blepharospasm disability index

BSDI is a validated tool for assessing functional impairments in daily activities^30^. It includes a total of six items (reading, driving, watching TV, shopping, doing daily activities, and walking), and each item is rated on a scale from 0 (no impairment) to 4 (not possible), with a ‘not applicable’ option. The BSDI score is the mean score obtained by dividing the total score by the number of applicable items.

### Tear film lipid layer thickness

The tear film LLT was detected using the LipiView® II Ocular Surface Interferometer (TearScience, Inc., Morrisville, NC)^31^. The exact LLT value cannot be estimated precisely or shown by the machine if the value exceeds 100 nm, so we set the tear film LLT to 100 nm in cases where it exceeded 100 nm. The recorded LLT was recognized as the major index of meibomian gland function.

### Assessment of blink rates and patterns

The eyelid blink rate and pattern were automatically recorded and analysed by the LipiView® II interferometer (TearScience, Inc.)^32^. Blinks without complete eyelid closure (partial blink) were distinguished from complete blink. The partial blink ratio for each eye was recorded for 20 s.

### Grading of meibomian gland dropout

The structure of the meibomian glands in each eye was assessed using a near-infrared meibography camera incorporated in the LipiView® II interferometer (TearScience, Inc.). To minimize manipulation of the eyelids, only meibomian glands in the lower eyelids were examined. The severity of meibomian gland dropout was classified from degree 0 (0%) to degree 4 (>75%) according to the meiboscale proposed by Dr. Heiko in 2012^33^, where the degree increases by 1 for every 25% gland loss.

### Quantification of aqueous tear production

Schirmer test 1 (ST-1) was used to quantify aqueous tear production from each eye^34^. The Schirmer strip was suspended on the inferior eyelid, between the inner two-third and the outer one-third portions, for 5 min without topical anaesthesia. The length of the wetted part on the test strip was recorded.

### Testing of eyelid laxity

Eyelid laxity was assessed using three different methods: the snap-back test, the eyelid distraction test, and canthal laxity (including medial and lateral canthi) test^35^. The snap-back test was performed by pulling the lower eyelid downward for approximately 5 s and recording the time taken by the eyelid to return to the globe before another blink. An increased time indicated increased laxity. In the distraction test, the lower eyelid was grasped and pulled away from the globe, and the distance between the eyelid and the globe was measured. A distance greater than 6 mm was considered abnormal. The medial canthal laxity test was conducted by pulling the lower lid laterally from the medial canthus and measuring the displacement of the medial punctum. The lateral canthal laxity test was conducted by pulling the lower lid medially from the lateral canthus and measuring the displacement of the lateral canthal angle.

### Sample size determination

The sample size was calculated by a free online power analysis program (G*power, version 3.1.9.2, Heinrich-Heine-Universität Düsseldorf)^36^. Because no previous study has focused on the tear film LLT in patients with BEB or HFS, we estimated the sample size according to the change in the tear film break-up time after BoNT injection in the Kocabeyoglu study^37^. We adopted the significance level (α) as 0.05, the desired power (1-β) as 0.9, and the effect size 0.913. Accordingly, the estimated sample size was 15 eyes.

### Statistical analysis

All statistical analyses were performed by free online calculators, including GraphPad software (https://www.graphpad.com/quickcalcs/index.cfm) and Social Science Statistics (http://www.socscistatistics.com/tests/signedranks/Default2.aspx). All results are expressed as means ± standard deviations. The Mann–Whitney U test and Fisher’s exact test were used to compare the parameters for BEB and HES patients. The Wilcoxon signed rank test and McNemar’s test were used to compare parameters before and 1 month after BoNT injection. A general linear regression model was used to explore the parametric association between meibomian gland function and eyelid performance. A *P*-value of <0.05 was considered statistically significant [marked with asterisks (*)].

## Results

Between 1 February 2016 and 30 June 2016, a total of 17 patients (11 with BEB and 6 with HFS) who met the inclusion and exclusion criteria were enrolled in this study (Table 1). There were no significant differences in most parameters associated with meibomian gland function and eyelid functional performance between the BEB and HFS groups. However, before the injection of BoNT, BEB patients exhibited significantly lesser medial canthal laxity and more irritation symptoms than did HFS patients. All patients exhibited a high prevalence of dry eye (all BEB patients and two-third of the HFS patients) and a low mean Schirmer 1 value (<3 mm).

**Table 1:** Demographic data of patients with facial motor disorders.

	Total (17 eyes)	Blepharospasm (11 eyes)	Hemifacial spasm (6 eyes)	*P value* ^d^
Gender^a^				0.58
Male	4 (23.5)	2 (18.2)	2 (33.3)	
Female	13 (76.5)	9 (81.8)	4 (66.7)	
Age (yr)^b^	70.4 ± 9.0	67.3 ± 8.2	76.0 ± 8.1	0.08
BSDI (score)^b^	7.8 ± 4.5	9.3 ± 4.9	5.2 ± 1.7	0.10
Snap-back test (sec)^b^	1.2 ± 2.0	1.1 ± 1.9	1.5 ± 2.4	0.76
Distraction test (mm)^b^	4.8 ± 1.3	5.2 ± 1.3	4.2 ± 1.0	0.13
Canthal laxity (mm)^b^				
Medial	0.41 ± 0.51	0.18 ± 0.41	0.83 ± 0.41	**0.03** ^*^
Lateral	0.65 ± 0.70	0.82 ± 0.75	0.33 ± 0.52	0.23
Ocular symptoms^a^				
Dryness	15 (88.2)	11 (100)	4 (66.7)	0.11
Irritate	10 (58.8)	9 (81.2)	1 (16.7)	**0.03** ^*^
Tearing	9 (52.9)	5 (45.5)	4 (66.7)	0.62
LLT (nm)^b^	75.1 ± 21.2	67.8 ± 19.3	87.2 ± 20.0	0.09
MG dropout (grade)^b^	1.7 ± 0.7	1.7 ± 0.7	1.6 ± 0.7	0.80
Schirmer test 1 (mm)^b^	2.4 ± 2.0	2.6 ± 2.1	2.2 ± 1.8	0.65
Total blinks (times)^bc^	5.41 ± 3.34	6.18 ± 3.95	4.0 ± 0.89	0.11
Partial blink ratio (%)^bc^	39.3 ± 32.0	36.3 ± 36.1	45.0 ± 24.9	0.51

^a^The categorical variants were showed in number (%); ^b^The continuous variants were showed in mean ± SD; ^c^Total blinks are defined by total times of blinks recorded during the 20-s assessment, while partial blink ratio is the percentage of the times of incomplete blinks during the same 20-s assessment divided by total blinks; ^d^Statistical test by Mann-Whitney U test and Fisher exact test, *P* < 0.05 was recognized statistically significant (^*^). Abbreviation: BSDI, blepharospasm disability index; LLT, lipid layer thickness; MG, meibomian gland.

**Figure 2:**
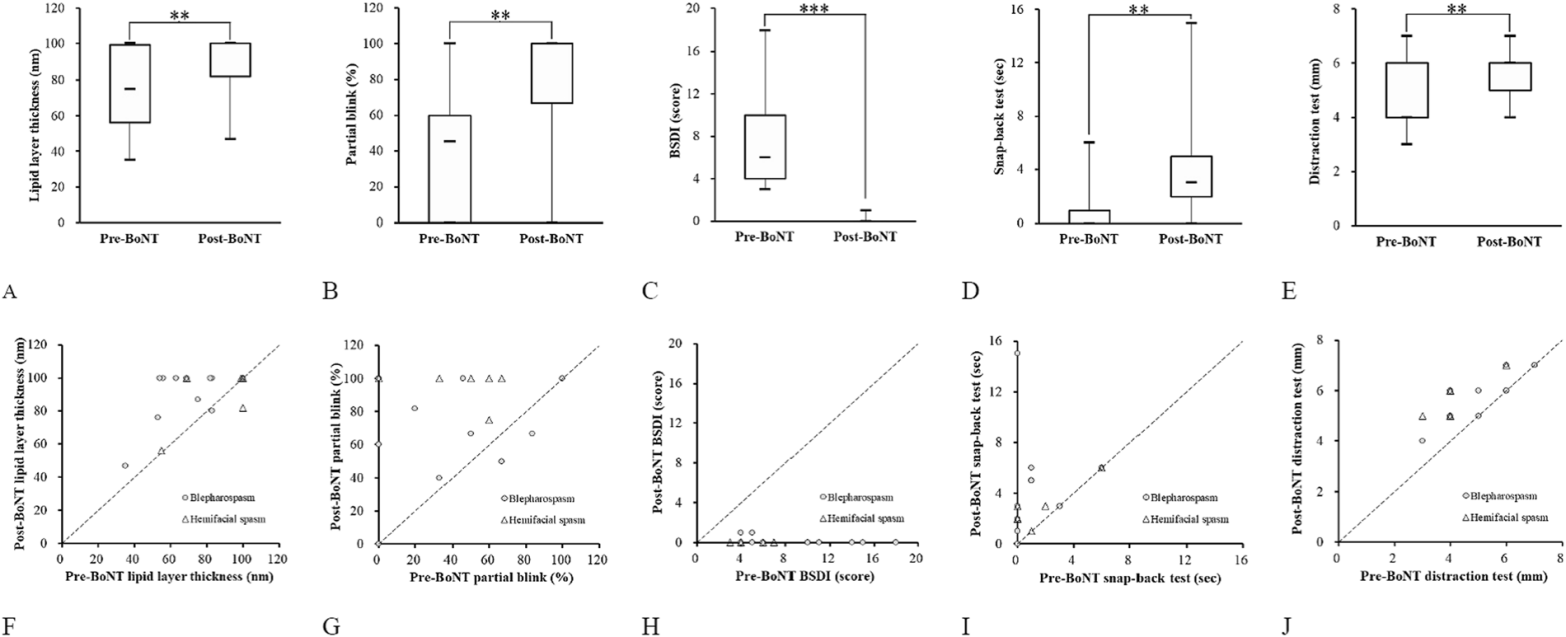
The performance indices with statistically significant changes after injection of botulinum neurotoxin. (**A–E**) show the differences of the performance indices before and after injection of botulinum neurotoxin by means of boxplots. (**F–J**) show the changes of the performance indices after injection of botulinum neurotoxin by means of scatter plots. BoNT, botulinum neurotoxin (**A**) BSDI, blepharospasm disability index; Partial blink, the ratio of incomplete blinks divided by total blinks during the same 20-s assessment.

All 17 patients completed pre-treatment and 1-month follow-up examinations (Table 2). Dryness was significantly decreased after BoNT injection. Both LLT and tear production (Schirmer 1) showed an increase, although the change was significant only for LLT (76.2 nm to 90.0 nm; *P* = 0.01). All patients felt significantly improved in the frequency and intensity of spasm (BSDI). Among the eyelid performance indices, including the partial blink ratio, the snap-back time, the distraction distance, and lateral canthal laxity, showed significant increase after BoNT injection. The total blink rate decreased 1 month after BoNT injection, but there was no statistical significance. The parameters with highly significant changes are shown in Fig. 2(A–E). These parameters showed similar changes in both the BEB and HFS groups (Fig. 2F–J).

**Table 2:** Changes of clinical parameters 1 month after botulinum toxin injection for all 17 patients with facial motor disorders.

	Before injection	1 month after injection	*P value* ^*d*^
Ocular symptom^a^			
Dryness	15 (88.2)	5 (29.4)	**0.004** ^*^
Irritation	10 (58.8)	5 (29.4)	0.07
Tearing	9 (52.9)	8 (47.1)	1.00
LLT (nm)^b^	75.1 ± 21.2	89.9 ± 16.7	**0.01** ^*^
MG dropout (grade)^b^	1.7 ± 0.7	1.7 ± 0.8	1.00
Schirmer test 1 (mm)^b^	2.4 ± 2.0	3.9 ± 4.3	0.35
Total blinks (times)^bc^	5.41 ± 3.34	4.53 ± 2.63	0.23
Partial blink ratio (%)^bc^	39.3 ± 32.0	78.8 ± 28.8	**0.003** ^*^
BSDI^b^	7.8 ± 4.5	0.12 ± 0.33	**<0.001** ^*^
Snap-back test (sec)^b^	1.2 ± 2.0	3.8 ± 3.4	**0.002** ^*^
Distraction test (mm)^b^	4.8 ±1.3	5.8 ± 0.9	**0.002** ^*^
Canthal laxity (mm)^b^			
Medial	0.41 ± 0.51	0.41 ± 0.51	1.00
Lateral	0.65 ± 0.70	1.18 ± 0.53	**0.02** ^*^
Lagophthalmos^a^	0 (0)	2 (11.8)	0.48
Blepharoptosis^a^	0 (0)	1 (5.9)	1.00
Ectropion^a^	1 (5.9)	2 (11.8)	1.00
Corneal erosion^a^	3 (17.6)	2 (11.8)	1.00

^a^The categorical variants were showed in number (%); ^b^The continuous variants were showed in mean ± SD; ^c^Total blinks are defined by the total times of blinks recorded during the 20-s assessment, while partial blink ratio is the percentage of the times of incomplete blinks during the same 20-s assessment divided by total blinks; ^d^Statistical test by Wilcoxon signed rank test and McNemar test, *P* < 0.05 was recognized statistically significant (^*^). Abbreviation: LLT, lipid layer thickness; MG, meibomian gland; BSDI, blepharospasm disability index.

**Figure 3:**
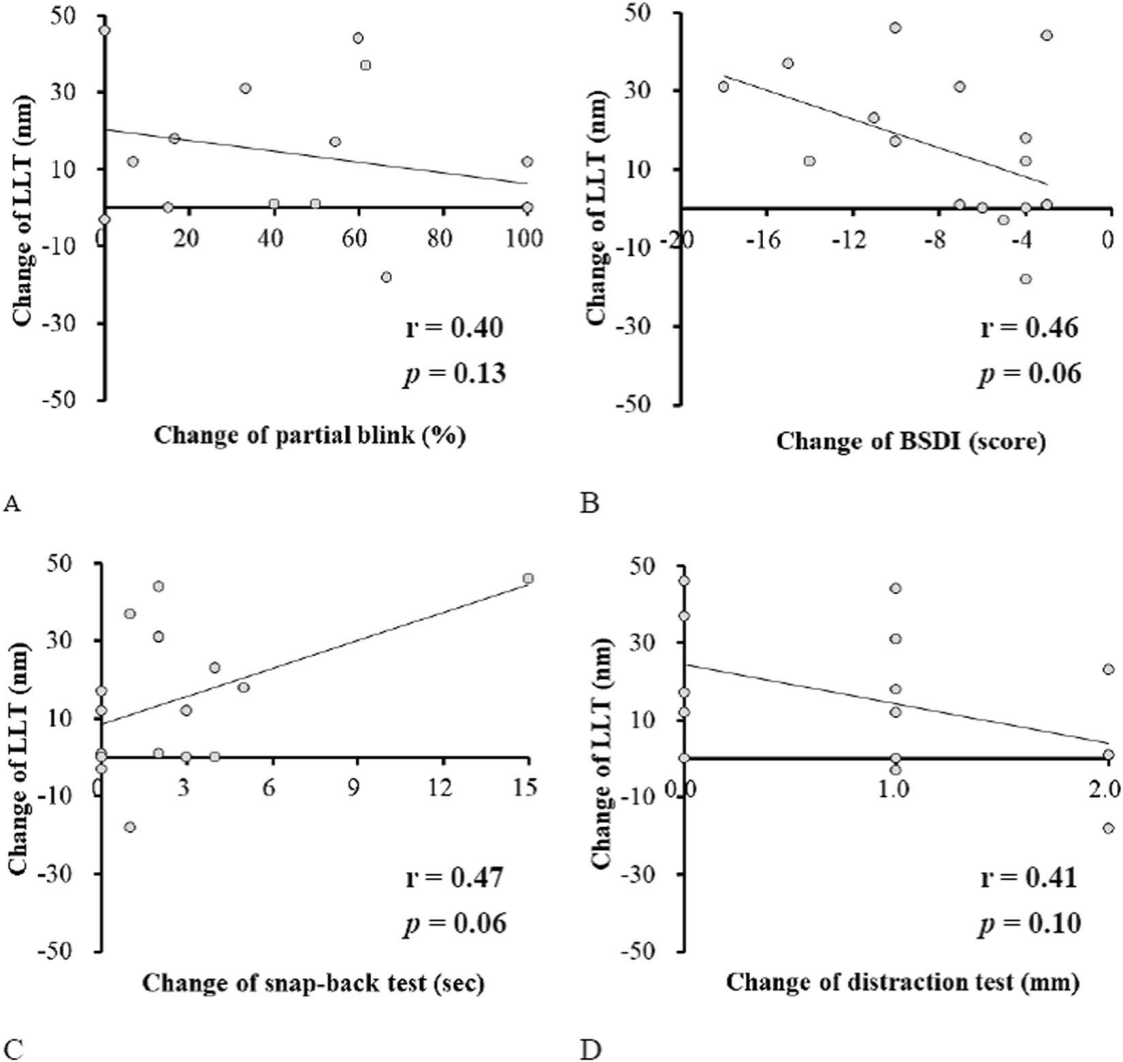
The associations between change of lipid layer thickness and changes of the potential indices of eyelid performance after injection of botulinum neurotoxin. (**A**) the correlation between change of partial blink change of lipid layer thickness. (**B)** the correlation between change of blepharospasm disability index and change of lipid layer thickness. (**C**) the correlation between change of snap-back test and change of lipid layer thickness. (**D**) the correlation between change of distraction test and change of lipid layer thickness. LLT, lipid layer thickness; BSDI, blepharospasm disability index; BoNT, botulinum neurotoxin A.

The association between changes in LLT and changes in the potential eyelid performance indices after BoNT injection were further analysed (Fig. 3). Although no significant association was noted, the association of the increased LLT with the decreased BSDI score and increased snap-back time was considerably strong. Therefore, we performed stepwise linear regression analysis to explore the association between LLT and all the indices combined and found that the increase in LLT could be better interpreted by a simultaneous decrease in the BSDI score and increase in the snap-back time [ΔLLT = −1.6 × (ΔBSDI) + 2.1 × (ΔSBT) − 3.2; r = 0.62 and *P* = 0.036; Δ = post-injection value minus pre-injection value].

Although medial canthal laxity and irritation symptoms were different between the BEB and HFS groups at baseline (Table 1), there was no significant change in the two parameters after BoNT injection in both groups. The overall post-treatment complication rate was 23.5% (four of 17 eyes; Table 2); the complications included mild lagophthalmos (n = 2, space <1.0 mm, 11.7%), mild blepharoptosis (n = 1, 5.9%), and newly developed ectropion (n = 1, 5.9%). Three eyes (17.6%) exhibited mild corneal punctate lesions before BoNT injection, which disappeared at 1 month after treatment in one of the three eyes. Therefore, no BoNT-related punctate keratopathy occurred in this study.

## Discussion

Dry eye disease is the most common complication of BoNT injection for facial movement disorders^19^. Meibomian gland dysfunction is now recognized as the major cause of dry eye disease. However, to our knowledge, no previous study has explored changes in meibomian gland function by measuring the tear film LLT after BoNT injection. In the present study, we found that aqueous tear deficiency was common, both before and after BoNT injection, in patients with facial movement disorders. Surprisingly, dry eye symptoms were alleviated after BoNT injection in our patients, with the possible mechanism being an increase in the tear film LLT associated with a simultaneous decrease in the BSDI score and increase in the snap-back time. Accordingly, we proposed that BoNT may improve meibomian gland function before it loses its anti-spasm actions, thus providing temporary relief from dry eye symptoms.

A previous review showed that 75–100% (mean, 93.3%) BEB patients in 29 studies exhibited relief from spasms after BoNT injection^24^. Similarly, all BEB and HFS patients in the present study experienced relief from spasms after BoNT injection, with a decrease in the mean BSDI score from 7.82 to 0.07 at 1 month after injection. In addition, as observed in previous reports^2,22,38^, the partial blink rate and eyelid laxity (snap-back test, distraction test, and lateral canthal laxity) also exhibited a significant increase after BoNT injection (Table 2). These changes can be attributed to the chemodenervation effect of BoNT on the orbicularis muscles, with pre-existing horizontal laxity of supportive structures (particularly the lateral canthal tendon) in the lower eyelid^2^. In addition to these well-known changes, we found that LLT increased after BoNT injection in BEB and HFS patients. Although the association of the LLT increase with the BSDI score decrease and snap-back time increase did not reach statistical significance (*P* = 0.06 for both; Fig. 3) in univariate analysis, the increase in LLT was significantly associated with a simultaneous decrease in the BSDI score and increase in the snap-back time in multivariate analysis.

The human meibomian glands are believed to be regulated by the cholinergic parasympathetic nervous system^39,40^. BoNT inhibits the release of acetylcholine from parasympathetic nerve endings and subsequently decreases lipid production. The acini, connecting ductiles, and central duct terminal of a meibomian gland are surrounded by the pretarsal orbicularis muscle and Riolan’s muscle, which compress these structures to result in meibum expression from the orifice into the tear film. BoNT paralyses these muscles and eventually decreases meibum excretion onto the ocular surface. Thus, BoNT should theoretically cause both secretory and excretory decreases in meibum and consequently reduce the tear film LLT. Ho *et al*. supported this inference and reported a decrease in the tear film stability, assessed by the tear film break-up time (TBUT) after BoNT injection for lateral canthal rhytids^16^. In contrast, Park *et al*. reported that TBUT significantly increased with an increased tear meniscus height at 2 weeks after BoNT injection^26^; furthermore, both Gunes *et al*. and Kocabeyoglu *et al*. found that TBUT was significantly higher at 3 weeks after injection^37,41^. We focused on the tear film LLT, which can reflect the tear film stability in an alternative manner and directly reveal meibomian gland function^42^, and found that BoNT injection significantly increased the tear film LLT without affecting meibomian gland dropout (Table 2).

Ho *et al*. found BoNT injection in the lateral canthal region caused persistent decrease of TBUT for 3 months and significant reduction of ST-1 at one month after treatment^16^. On the contrary, Sahlin *et al*. found BoNT injection into the medial part of the eyelids decreased the lacrimal drainage, suggesting an alternative way to treat dry eye disease^43^. Yang *et al*. further found patients having BoNT injection at the medial lower eyelid had better results of TBUT and ST-1 than those not at the medial lower eyelid^44^. Both studies mentioned above suggested BoNT injection at the medial eyelid, especially at the medial lower eyelid associated with predominant punctum drainage, paralyzes pre-tarsal orbicularis oculi muscle and makes the puncta and the canaliculi loosening, which results in poor pumping force of the lacrimal drainage system and tear retention. Besides, BoNT causes flaccid eyelids impairing the apposition of the puncta, which might worsen the drainage efficacy. Our injection sites included the loci at the medial pre-tarsal orbicularis oculi muscle, just inferior to lower lacrimal punctum (Fig. 1). Therefore, the massive paralytic effects in the medial canthal region should outweigh the inhibitory effects of BoNT injection on meibum secretion and excretion from affected glands. Multivariate regression analysis in the present study showed that the combination of a decreased BSDI score and an increased snap-back time could further explain the increase in LLT after BoNT injection. This result further demonstrated that the mechanism underlying the increase in LLT after BoNT injection was probably paralysis of the orbicularis muscle, which resulted in decreased pumping of the lacrimal drainage system.

Dry eye is a common side effect of BoNT treatment, with a reported incidence rate of up to 70%^19^. Chemode-nervation of the orbicularis muscle with lagophthalmos, ectropion, and abnormal blinks lead to corneal exposure and ocular surface desiccation. In the present study, there was no statistically significant change in the Schirmer 1 value after BoNT injection (2.4 ± 2.0 s vs. 3.9 ± 4.3 s), although the patients experienced significant relief from dryness (Table 2). This relief could be attributed to the increase in LLT in association with a simultaneous decrease in the BSDI score and increase in the snap-back time. However, future studies focused on the correlation between specific dry eye symptoms (using dry eye questionnaires) and comprehensive tear parameters in patients with BEB and HFS are necessary.

According to the definition of BEB subgroups in Ferrazzano *et al*.^45^, our BEB patients included 7 clonic spasms and 4 tonic spasms. Ferrazzano found only clonic spasms rather than tonic spasms had reduced blink rates after BoNT injection, while we did not find significant reduced blink rates (total blinks) after BoNT injection in both spasm types (*P* = 0.207 for clonic spasms; *P* = 0.125 for tonic spasms). Interestingly, we found partial blink ratio in clonic spasms was statistically increased after BoNT injection (Pre- *vs*. Post-BoNT: 35.54 ± 41.97% *vs*. 86.93 ± 17.54%, *P* = 0.046), but there was no difference in tonic spasms (37.50 ± 28.46% *vs*. 39.17 ± 28.33%, *P* = 0.785). Similar to the assumption of Ferrazzano, we found patients with clonic spasms had lower baseline ST-1 than those with tonic spasms with marginal difference (1.57 ± 1.40 mm vs. 4.25 ± 2.22 mm, *P* = 0.068), while there was no difference between these 2 types after BoNT injection (3.29 ± 2.43 mm *vs*. 2.50 ± 3.70 mm, *P* = 0.21). Therefore, our results suggested that increased partial blink ratio rather than reduced blink rates resulted in lacrimal drainage reduction and following tear retention in clonic BEB patients after BoNT injection. However, the case number was too small in both types of BEB, a further large-scale study will be needed to confirm this implication.

This study has few limitations. First, the follow-up period was relatively short because the effects of BoNT could last for several months. However, our study focused on the maximal effect of BoNT on LLT and tear production, which is typically seen within 1 month of injection. Further studies are necessary to verify the long-term effects of BoNT treatment on meibomian gland function. Second, the previous effects of BoNT may not have totally diminished before we administered another injection. Previous studies have shown that the clinical benefits of BoNT usually last for 3–4 months in most patients, but they can also last for 6 months or longer^2^. In our study, all patients received injections at intervals of at least 4 months, and they had already complained of difficulty in opening their eyes, which strongly affected their daily life, before BoNT treatment.

In conclusion, BoNT injection in patients with BEB and HFS can provide immense relief from spasms. LLT, a decisive factor for tear film stability, may show a significant increase at 1 month after injection in both BEB and HFS patients. A simultaneous decrease in the BSDI score and increase in the snap-back time could be a contributing factor to the increase in LLT via decreased lacrimal drainage; this is probably the mechanism underlying the relief from dryness after BoNT injection in patients with facial movement disorders.
